# Intact Transmastoid Ossicle Swaying Technique to Preserve Hearing in Pediatric Facial Nerve Decompression Surgery: A Case Report

**DOI:** 10.7759/cureus.58269

**Published:** 2024-04-14

**Authors:** Masao Noda, Ryota Koshu, Mari Dias, Ryotaro Onaga, Makoto Ito

**Affiliations:** 1 Otolaryngology - Head and Neck Surgery, Jichi Medical University, Shimotsuke, JPN; 2 Otolaryngology - Head and Neck Surgery, Jichi Medical University Hospital, Shimotsuke, JPN

**Keywords:** pediatric otolaryngology, ear surgery, surgical technique, hearing preservation, facial palsy, facial nerve decompression

## Abstract

When pharmacological treatments are inadequate, facial nerve paralysis from various etiologies, including Bell’s palsy, Hunt syndrome, and trauma, often requires surgical intervention. Facial nerve decompression surgery aims to relieve nerve compression and restore function, with preserving hearing function, especially in pediatric cases, being crucial. Conventional methods, like the transmastoid approach, risk affecting auditory function due to ossicle manipulation. Herein, we describe the case of a 12-year-old boy with left facial palsy diagnosed with zoster sine herpete (ZSH) syndrome. Despite medical treatment, the patient’s condition did not improve, prompting facial nerve decompression surgery. Employing the intact transmastoid ossicle (ITO) swaying technique, we minimized ossicular manipulation, preserving auditory function while effectively achieving facial nerve decompression. The patient demonstrated improvement postoperatively in auditory and facial nerve functions. Furthermore, audiometric assessments demonstrated no substantial deterioration in hearing thresholds, and the facial nerve function improved from Grade V to Grade II on the House-Brackmann scale. The ITO technique provides a less invasive alternative compared to conventional approaches, lowering the chance of the ossicular chain and the risk of postoperative hearing loss. This case highlights the significance of customized surgical approaches in pediatric facial nerve decompression surgery, resulting in improved patient outcomes. Further research is required to validate the efficacy and safety of this method across various clinical contexts.

## Introduction

Facial nerve paralysis, originating from various conditions, such as Bell’s palsy, Hunt syndrome, or trauma, presents a diverse etiology spectrum [1-3]. While several cases may benefit from pharmacological interventions, such as prednisolone or antiviral medications, severe cases often require surgical intervention, particularly facial nerve decompression [4-6]. This procedure aims to relieve facial nerve compression by removing the surrounding bone within the temporal bone, enabling constricted nerve release and recovery [6,7].

The transmastoid approach is a frequently used method for facial nerve decompression, spanning from the geniculate ganglion to the mastoid portion, and is relatively minimally invasive [8-11]. However, preserving auditory function can be challenging with this approach due to the need to reposition the ossicles to secure the surgical site, which can result in complications, such as hearing disorders [10,11].

Recent developments, like transcanal endoscopic ear surgery (TEES), present a less invasive option, where the middle ear surgery is performed through the ear canal using an endoscope [12-14]. However, TEES, while reducing invasiveness, has limitations in its scope of operation and visual confirmation capabilities.

Alternatively, Inagaki et al. reported decompressing the facial nerve using microscopy via the transmastoid approach without removing the ossicles [15]. If ossicles can be securely swayed aside, this technique offers a broader and more manageable operating field without compromising stability, thus improving the effectiveness in adult Bell’s palsy cases.

This report provides details of a pediatric patient with severe facial palsy treated using facial nerve decompression without ossicle repositioning, but rather by swaying them forward after partial incus relocation. We assessed the efficacy of this technique for postoperative facial nerve function and hearing preservation using fibrin glue. The intact transmastoid ossicle (ITO) swaying technique introduces a novel approach to address this challenge by maintaining ossicle integrity while achieving facial nerve decompression.

## Case presentation

A 12-year-old boy exhibited left facial palsy and left ear. Initial examination at a primary care clinic revealed severe facial palsy and erythema of the left tympanic membrane, raising the suspicion of left Hunt syndrome and prompting referral to our facility.

**Figure 1:**
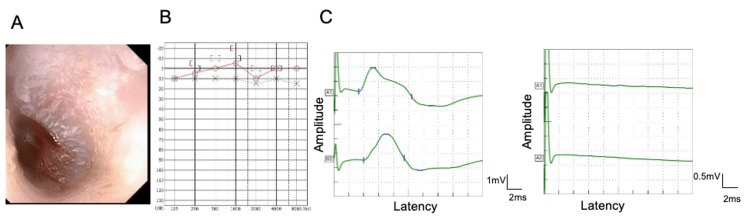
Physical findings of the patient at our department. A: Ear endoscopy demonstrating erythema of the tympanic membrane. B: Pure-tone audiometry showing a normal hearing level. C: The electroneurography shows a response on the right side (left side of the screen) but not on the left side (right side of the screen). The upper and lower waves represent waveforms obtained from orbicularis oculi and orbicularis oris, respectively.

Upon presentation to our hospital, the vesicles were observed in the posterior part of the left ear alongside the erythema of the tympanic membrane (Figure 1A). Left facial nerve paralysis was assessed using the House-Brackmann grading system [16], resulting in a score of Grade V. According to the Yanagihara method [17], the score was 4/40. Audiometric assessment using the four-frequency average method revealed no significant air-bone gap, with average thresholds of 2.5 dB on the right and 5.0 dB on the left using the four-frequency average method (Figure 1B).

Serological analysis revealed high VZV IgG antibody levels (1200 IU/ml), indicating zoster sine herpete (ZSH) syndrome. The patient was hospitalized and treated with intravenous prednisolone (2 mg/kg/d, tapered over 10 d) and oral valacyclovir (3000 mg/d for seven days).

Electroneurography was performed for prognostic evaluation 12 days post-onset, which yielded a result of 0% on the left side with up to 40 mA of stimulation (Figure 1C). CT scan showed no obvious abnormalities in the brain, middle ear, or parotid gland. Due to no improvement in facial movement, the decision to proceed with facial nerve decompression surgery was made.

Surgical procedure

**Figure 2:**
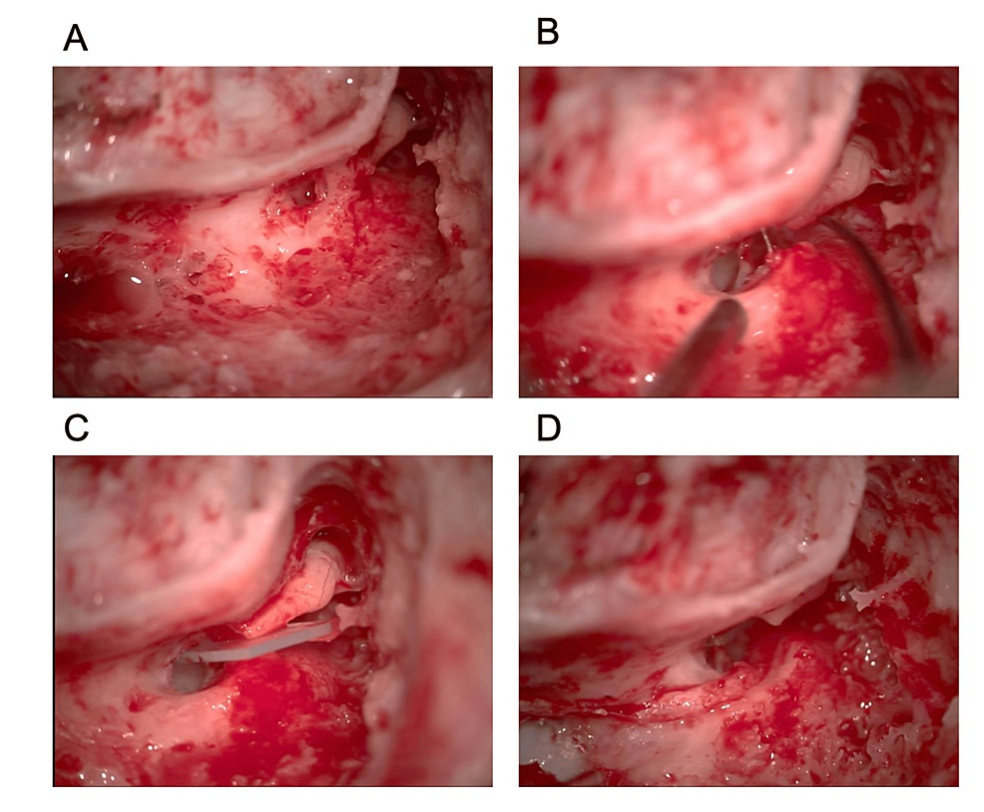
Surgical procedure of the facial nerve decompression. After the posterior tympanotomy (A), the incus–stapes joint was disconnected and the incus was swayed to the posterior wall of the external auditory canal (B). After placing a silicone sheet between the stapes and incus (C), the facial nerve was exposed from the geniculate ganglion to the horizontal and vertical portions of the digastric ridge (D).

A posterior superior auricular skin incision was made, followed by mastoidectomy. The incus and digastric ridge were located, and a posterior tympanotomy was performed to open the posterior tympanic cavity (Figure 2A). Subsequently, the incus-stapes joint was gently dislocated, and the incus body part was displaced outward. Next, the fibrin glue was used to adhere it to the posterior wall of the external auditory canal (Figure 2B). A silicone sheet was placed in between to facilitate facial nerve manipulation and exposure (Figure 2C). The facial nerve was exposed from the vertical segment to the second genu and the geniculate ganglion was identified.

Bulging of the nerve was observed when the nerve sheath was incised. The incus returned to its original position from the posterior wall of the external auditory canal. Dexamethasone-impregnated gelfoam® (Pfizer) was placed around the exposed facial nerve, followed by the closure of the subcutaneous and cutaneous layers with sutures (Figure 2D).

Postoperative course

**Figure 3:**
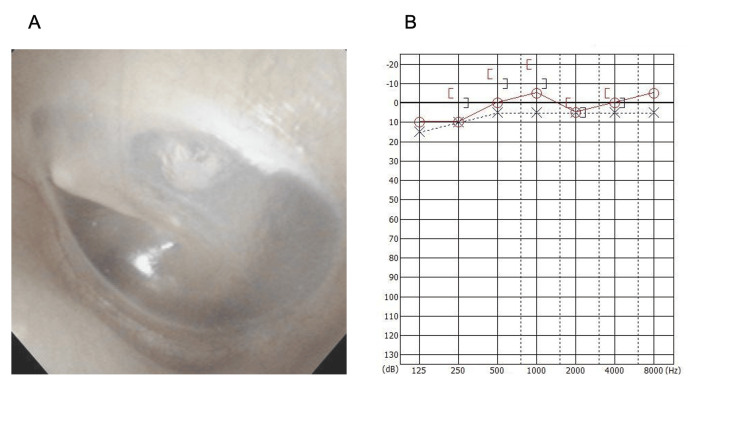
Postoperative findings Erythema of the tympanic membrane improved (A), and no postoperative worsening of hearing was observed (B).

The postoperative course was uneventful, and the patient was discharged on the fifth postoperative day. Auditory function was within the normal range at the 30-day follow-up and the function of the facial nerve improved to House-Brackmann Grade IV, with a score of 14/40 according to the Yanagihara method (Figure 3A, 3B).

At a follow-up examination five months postoperatively, the tympanic membrane showed normal appearance, auditory function remained stable, and facial nerve function substantially improved to House-Brackmann Grade II, with a score of 40/40 according to the Yanagihara method, indicating substantial improvement.

## Discussion

This study presents an ITO technique for facial nerve decompression in a pediatric patient. Facial nerve decompression surgery is vital for severe facial palsy cases, especially when drug treatment proves ineffective. In this study, a 12-year-old boy presented with left facial palsy and left ear pain, raising the suspicion of ZSH syndrome. Despite initial medical management with prednisolone and valacyclovir, the lack of improvement in the function of the facial nerve necessitated surgical intervention.

Facial nerve decompression surgery is necessary, particularly in severe facial palsy cases, despite its low frequency in pediatric patients [18]. The extent of functional and aesthetic impairments that these children exhibit is profoundly distressing for the patients and their parents. This highlights the urgency for prompt attention, treatment, and functional restoration. While Hunt syndrome is the second most prevalent non-traumatic cause of facial paralysis, its occurrence in children is relatively rare compared to adults [19]. If left untreated, conditions like Hunt syndrome or ZSH can lead to a poor prognosis, making surgical intervention the only viable option after pharmacological treatments.

A main advantage of the ITO technique is its ability to minimize trauma to the ossicular chain, thereby reducing the hearing loss risk, which is a common complication associated with conventional approaches involving ossicle repositioning. This technique ensures optimal auditory outcomes by preserving the ossicles’ anatomical integrity, particularly in pediatric patients, in whom preserving hearing function is paramount for language development and quality of life [20].

Anatomically, the ITO method offers considerable advantages. The distance between the fossa incudis and tympanic membrane (chorda tympani) determines the distance over which sway is achievable. Therefore, preoperative CT imaging to measure the spacing between the incus and the horizontal portion of the facial nerve, as well as the distance between the ossicles and tympanic membrane, proved to be effective. Repositioning the ossicles forward enhances visualization and maneuverability during surgery, enabling precise instrument manipulation with a margin of error as small as 1 mm. This approach also mitigates the risk of sensorineural hearing loss linked to complete ossicle removal. Furthermore, it facilitates access to critical nerve segments like the facial nerve genu and the tympanic portion.

Repositioning or completely removing the ossicles carries a risk of hearing loss. Elderly patients or those with ossicular stiffness due to chronic inflammation are especially vulnerable to the potential risk of inner ear damage when swaying the ossicles. The ITO technique involves careful manipulation of the ossicles, swaying them aside rather than disarticulating them, to access the facial nerve without jeopardizing auditory function. The success of the surgical procedure was confirmed by the postoperative outcomes. Notably, the patient demonstrated improvements in auditory and facial nerve functions. Audiometric assessments revealed no considerable deterioration in hearing thresholds, confirming the successful preservation of auditory function. Moreover, facial nerve function improved from Grade V to Grade II on the House-Brackmann scale, indicating substantial recovery of facial motor function.

Although the present case demonstrated promising results, acknowledging the limitations of this study is crucial. Being a single case report, the generalizability of the findings may be limited. Further research involving larger cohorts is necessary to validate the efficacy and safety of this technique in pediatric facial nerve decompression surgery.

In conclusion, the ITO technique is valuable for facial nerve decompression while maintaining hearing, especially in pediatric patients. This case underscores the significance of innovative surgical approaches tailored to individual patient needs, resulting in improved outcomes. Further studies are required to ascertain the long-term effectiveness and applicability of this technique in broader clinical contexts.

## Conclusions

The ITO technique shows promise in preserving hearing during pediatric facial nerve decompression surgery by minimizing ossicular manipulation, thus reducing the risk of sensorineural hearing loss.

This technique effectively achieves facial nerve decompression while preserving auditory function, offering a valuable addition to surgical options for pediatric facial palsy. Further research is required to validate its broader applicability and long-term outcomes.
